# Delayed Radial Nerve Recovery After Ballistic vs. Blunt Humeral Shaft Fractures: A Time-to-Recovery Analysis of Nerves in Continuity

**DOI:** 10.3390/jcm15197603

**Published:** 2026-09-30

**Authors:** Abdel Kareem Hilo, James Ha, Tyler Forbes, David Abdelnour, Magd Boutany, Bashar Jawich, Hadi Elmenini, Jawad Saad, Rebecca Schultz, Ameen Suhrawardy, Serge Andreou, Rahul Vaidya

**Affiliations:** 1 School of Medicine, Wayne State University, Detroit, MI 48201, USA; 2 Department of Orthopaedics, Detroit Medical Center, Detroit, MI 48201, USArahvaidya2012@gmail.com (R.V.)

**Keywords:** radial nerve palsy, humeral shaft fracture, gunshot wound, ballistic injury, nerve recovery, time-to-event analysis, Kaplan–Meier analysis, peripheral nerve injury

## Abstract

**Background/Objectives:** Radial nerve palsy complicates 8–16% of humeral shaft fractures, and because more than 90% of primary palsies recover spontaneously, the clinically decisive question is not whether a nerve will recover but when. That timeline has been defined almost entirely from blunt-mechanism cohorts, yet is routinely applied to ballistic injury. No study has directly compared radial nerve recovery between gunshot wound (GSW) and blunt trauma. We compared time to full radial nerve recovery between mechanisms within a cohort of radial nerve injuries with continuity either operatively confirmed or clinically inferred, so that injury mechanism was not confounded with complete transection. **Methods:** This retrospective cohort study was conducted at a single urban Level I trauma center between January 2000 and December 2023. Of 228 patients screened, 22 were excluded for age under 18 years, inadequate documentation or follow-up, or surgically confirmed radial nerve transection, leaving 206 patients (117 GSW, 89 blunt) containing 39 radial nerve injuries. Recovery was defined as the first recorded occurrence of full extension of the forearm, wrist, and digits. Kaplan–Meier estimation, log-rank testing, and Cox proportional hazards models were used, with covariate stratification, single-covariate adjustment, and sensitivity analyses restricted to operatively confirmed nerves. **Results:** The analytic cohort comprised 39 in-continuity (29 with operatively confirmed continuity and 10 with continuity inferred clinically) radial nerve injuries (25 GSW, 14 blunt) with 34 recovery events. Full recovery did not differ between mechanisms (84.0% vs. 92.9%; *p* = 0.64). Recovery was significantly slower after a gunshot wound: Kaplan–Meier median of 14.3 months (95% CI 7.5–17.3) versus 6.9 months (95% CI 3.6–8.2); log-rank *p* = 0.002; hazard ratio of 0.31, 95% CI of 0.14–0.67, and *p* = 0.003. Cumulative recovery at 12 months was 43% after gunshot wound versus 93% after blunt trauma, and 11 of 21 gunshot wound recoveries occurred beyond 12 months compared with 0 of 13 blunt recoveries (*p* = 0.002). No adjustment attenuated the mechanism effect. **Conclusions:** Among humeral shaft fractures with radial nerves in continuity, the observed proportion of radial nerve recovery did not differ significantly between ballistic and blunt mechanisms, although recovery occurred on different timelines. The study is limited by its retrospective design and small analytic sample of 39 patients, which may limit the generalizability and statistical power of the findings. Nonetheless, decision points of approximately one year, derived from blunt-mechanism and surgical-repair cohorts, should not be transferred unmodified to ballistic injury.

## 1. Introduction

Humerus fractures are commonly encountered by orthopedic trauma surgeons and account for a substantial proportion of upper extremity fractures each year [1]. In U.S. urban Level I trauma centers, humerus fractures result from a variety of mechanisms, including falls, motor vehicle collisions, and penetrating trauma such as gunshot wounds (GSW) [2,3,4]. Humerus fractures are often associated with extensive soft tissue damage, infection risk, vascular injury, and neurologic injury [5,6]. Among these complications, radial nerve injury is of particular concern due to the nerve’s close anatomic relationship to the humerus within the radial groove. As a result, humerus fractures place the radial nerve at risk, with rates of injury reported at 8–16% in closed fractures and substantially higher following ballistic injury [7,8,9].

The prognosis for radial nerve recovery is generally favorable, with more than 90% of primary radial nerve palsies recovering spontaneously without surgical intervention [7,8,10]. Given this injury’s prognosis, the clinically decisive question is not whether a nerve will recover but when. The decision to explore, graft, or proceed to tendon transfer must be made before the eventual outcome is observable, and is therefore decided based on how much time has elapsed without adequate recovery [11,12]. In the literature, however, that timeline has been defined almost entirely from blunt-mechanism cohorts, and the definition of “recovery” varies across studies. Very few studies look at the timing to reach full recovery in a GSW exclusive cohort [5,13]. Entezari et al. reported a median of 7 to 9 weeks to the first documented sign of recovery after blunt humeral shaft fracture and recommended nerve studies if no sign of recovery was evident by 9 weeks, an onset-based endpoint [8]. Ostermann et al. used a full-recovery endpoint defined as complete return of manual strength. They found that nearly all patients who recover do so within 52 weeks, and that injury mechanism, fracture type, location, and treatment do not influence recovery. That analysis, however, contrasted mechanisms within lower-grade palsies and did not include ballistic injury [14]. Krijnen et al. modeled only a modest residual probability of recovery, approximately 17%, among patients not yet recovered at one year following surgical repair [15]. These studies evaluate different stages of recovery and are therefore not interchangeable endpoints: Entezari et al. dated the first sign of recovery, Ostermann et al. assessed complete motor recovery, and Krijnen et al. examined recovery after surgical nerve repair. The present study differs by evaluating time to full motor recovery in nerves confirmed or clinically inferred to be in continuity, without surgical nerve repair, and directly comparing ballistic with blunt mechanisms. These estimates have been used to inform expectations for ballistic injuries despite differences in mechanism and recovery endpoint. Prior comparative studies have contrasted only high- versus low-energy blunt mechanisms [14], and the single study that directly compared gunshot wound with blunt radial nerve injury examined electrodiagnostic lesion characteristics rather than recovery [16]. No study has directly compared radial nerve recovery between gunshot wound and blunt trauma: the two most common mechanisms of humeral shaft fracture in the urban trauma setting.

While radial nerve injury results from both blunt and penetrating trauma, the mechanism of injury to the nerve differs. Blunt trauma typically produces indirect nerve injury from fracture displacement or traction, whereas penetrating trauma may cause more direct neural damage through projectile impact, cavitation, and associated soft tissue disruption [8,15,16,17]. One possible biological explanation for the delayed recovery observed after ballistic injury is that temporary cavitation and transmitted pressure waves may produce a broader zone of axonal injury beyond the projectile track. Because axonal outgrowth after fracture-associated radial palsy proceeds at roughly 1 mm per day, a greater extent of axonal injury could theoretically translate into a longer time to complete reinnervation even when the nerve remains anatomically intact. This represents a potential mechanism for the observed difference in recovery timing, rather than a mechanism directly demonstrated by the present study [18,19,20].

The primary purpose of this study was to compare the time to full radial nerve recovery between gunshot wound and blunt trauma. Full recovery was defined as the first recorded occurrence of full active extension of the forearm, wrist, and digits. To ensure that injury mechanism was not confounded with complete transection, the cohort was restricted to nerves with continuity either operatively confirmed or clinically inferred. This head-to-head comparison of recovery timing between mechanisms in non-transected nerves has not previously been performed. The secondary aims were to report injury characteristics between mechanisms stratified by management. By dividing outcomes across both injury mechanism and treatment status, and by applying time-to-event methods that account for variable follow-up, this study aims to better characterize the long-term clinical course of radial nerve recovery after humeral shaft fracture.

## 2. Materials and Methods

### 2.1. Study Design and Patient Population

This retrospective cohort study was approved by the Institutional Review Board with a waiver of informed consent (protocol 21-10-4123) approved on 11 February 2025. Adults presenting to a single urban Level I trauma center in the United States with a humeral shaft fracture secondary to GSW or blunt trauma between January 2000 and December 2023 were identified by institutional chart review. Patients were excluded if they were younger than 18 years, had inadequate clinical documentation or follow-up, or had a surgically confirmed radial nerve transection. Inadequate follow-up was defined as absence of any postoperative or trauma-clinic follow-up within our health system after the index treatment, including patients whose subsequent care occurred exclusively at an outside institution for which follow-up records were unavailable. Of 228 patients screened, 22 were excluded: *n* = 16 for age < 18 years, *n* = 4 for inadequate documentation or follow-up, and *n* = 2 for surgically confirmed radial nerve transection, leaving a descriptive cohort of 206 patients (117 GSW, 89 blunt trauma). All 39 radial nerve injuries identified within this cohort were carried forward into the analytic cohort; no further exclusions were applied.

### 2.2. Definition of Recovery and Determination of Follow-Up

Radial nerve injury was defined by wrist drop, finger drop, or weakness of forearm extension documented on the initial presentation examination. All radial nerve injuries included in the analytic cohort were present at the time of the index injury; no patient developed a new radial nerve palsy after operative or nonoperative treatment. Radial nerve function was assessed clinically at follow-up using motor and sensory examination. Motor assessment included active extension of the forearm, wrist, and digits, while sensory examination evaluated sensation in the radial nerve distribution. Full radial nerve recovery was defined solely on clinical examination—the first documented restoration of full active extension of the forearm, wrist, and digits without residual motor deficit. Electrodiagnostic studies were not used to define or date the recovery endpoint. Time to recovery was calculated in months from injury to that visit.

No minimum follow-up threshold was imposed on the analytic cohort. Because time-to-event methods accommodate variable observation length by design, a fixed follow-up requirement would discard information without improving validity. Patients with inadequate clinical documentation or follow-up were excluded at screening before the descriptive cohort of 206 patients was defined, and it therefore never entered the survival analysis. Within the analytic cohort of 39 in-continuity radial nerve injuries, every patient had a documented final neurologic status and none were lost to follow-up. Thirty-four patients met the predefined recovery criterion and contributed a recovery event at the first visit at which full radial nerve recovery was documented. The remaining five patients had persistent radial nerve motor deficits without documented full recovery and demonstrated no meaningful clinical improvement during follow-up. Electrodiagnostic studies were interpreted as indicating a poor likelihood of further radial nerve recovery. Because the predefined event was documented full motor recovery and this event did not occur, these patients were right-censored at their final available neurologic assessment.

Following Entezari et al., follow-up length was not fixed by protocol but was defined by what the retrospective records contained. Patients were seen at routine postoperative and trauma clinic visits, typically every 2 to 4 weeks, with neurologic status recorded at each visit. All patients with radial nerve injury were referred to physical and occupational therapy. Neurologic status at the final recorded visit determined the outcome for every patient in the analytic cohort.

### 2.3. Ascertainment of Nerve Continuity

Continuity was established by direct operative inspection in all 29 operatively treated patients in the analytic cohort (19 GSW, 10 blunt). In each operative case the radial nerve was visualized during the surgical approach and documented in the operative report as grossly intact. Among the ten nonoperatively treated patients, continuity could not be surgically confirmed. In nonoperatively managed injuries, radial nerve continuity was inferred using MRI and electrodiagnostic testing together with the clinical examination. Electrodiagnostic testing in these cases was used only to support the inference of preserved continuity, not to grade lesion severity or to define recovery. These findings were used to support the absence of nerve transection and preservation of continuity. This inference is weaker than direct operative confirmation, and a prespecified sensitivity analysis restricted to operatively confirmed nerves is reported.

### 2.4. Covariates and Control of Confounding

The principal threat to the interpretation of a mechanism effect is that established predictors of poor radial nerve recovery, such as nerve transection, higher lesion grade, associated vascular injury, and fracture pattern or location, are themselves more common after ballistic injury. Associated vascular injuries were treated with direct repair and vessel shortening when feasible or with vein grafting when primary repair could not be performed without tension. Our design addresses the first of these by construction, since transected nerves are excluded at screening and continuity is operatively confirmed in 29 of 39 patients. The remaining covariates were addressed analytically.

Age, vascular injury of the affected limb, fracture location (proximal shaft, midshaft, or distal humerus, classified radiographically), polytrauma status, sex, and treatment strategy were abstracted for every patient. Categories were not mutually exclusive because fractures spanning more than one anatomic region were classified in each involved region. We prespecified two approaches. First, mechanism-stratified estimates were generated within the cohort of each covariate to establish whether the direction of the mechanism effect was preserved within. Second, because 34 recovery events cannot support a fully saturated model without overfitting, covariates were entered into Cox models one at a time alongside mechanism, and the stability of the mechanism coefficient across these models was assessed rather than a single multivariable estimate being treated as definitive; a model containing all three injury-severity covariates simultaneously is reported as exploratory. These results are reported in Section 3.4.

### 2.5. Statistical Analysis

Continuous variables are summarized as median with interquartile range (IQR) and categorical variables as counts and percentages. Between-group comparisons used the Mann–Whitney U test for continuous variables and the two-sided Fisher exact test for all categorical variables. The prespecified primary analysis was the comparison of time to radial nerve recovery between mechanisms in the analytic in-continuity cohort, assessed by Kaplan–Meier estimation with log-rank testing and by Cox proportional hazards regression. Hazard ratios below 1.0 indicate slower recovery. Three supporting analyses were prespecified: landmark cumulative recovery at 3, 6, 12, 18, and 24 months; the proportion of recoveries occurring beyond 12 months, compared by Fisher exact test; and restricted mean time to recovery over a 24-month horizon, which does not depend on the proportional hazards assumption. Sensitivity analyses restricted the cohort to operatively confirmed nerves and applied penalized-likelihood estimation to assess small-sample bias. The proportional hazards assumption was assessed using scaled Schoenfeld residuals. Significance was defined as *p* < 0.05. Analyses were performed using R v4.5 (R Foundation for Statistical Computing, Vienna, Austria) using the survival and coxphf packages.

## 3. Results

### 3.1. Cohort Characteristics

The descriptive cohort comprised 206 patients and was 81.1% male. GSW patients were markedly younger (median of 28 [IQR 22–37] vs. 44 [29–62] years) and more often male (95.7% vs. 61.8%; *p* < 0.001). Operative fixation was performed in 127 patients (61.7%). Any neurologic injury was more common after GSW (35.9% vs. 19.1%; *p* = 0.009), and radial nerve injury was numerically more frequent after GSW (25/117, 21.4% vs. 14/89, 15.7%; *p* = 0.37). Vascular injury was more frequent after GSW (10.3% vs. 3.4%; *p* = 0.10). Fracture location did not differ significantly between mechanisms, although proximal shaft fractures were numerically more common after blunt trauma (43.8% vs. 33.3%; *p* = 0.15). Complications were uncommon and did not differ between mechanisms (Table 1).

### 3.2. The Analytic In-Continuity Cohort

**Figure 1:**
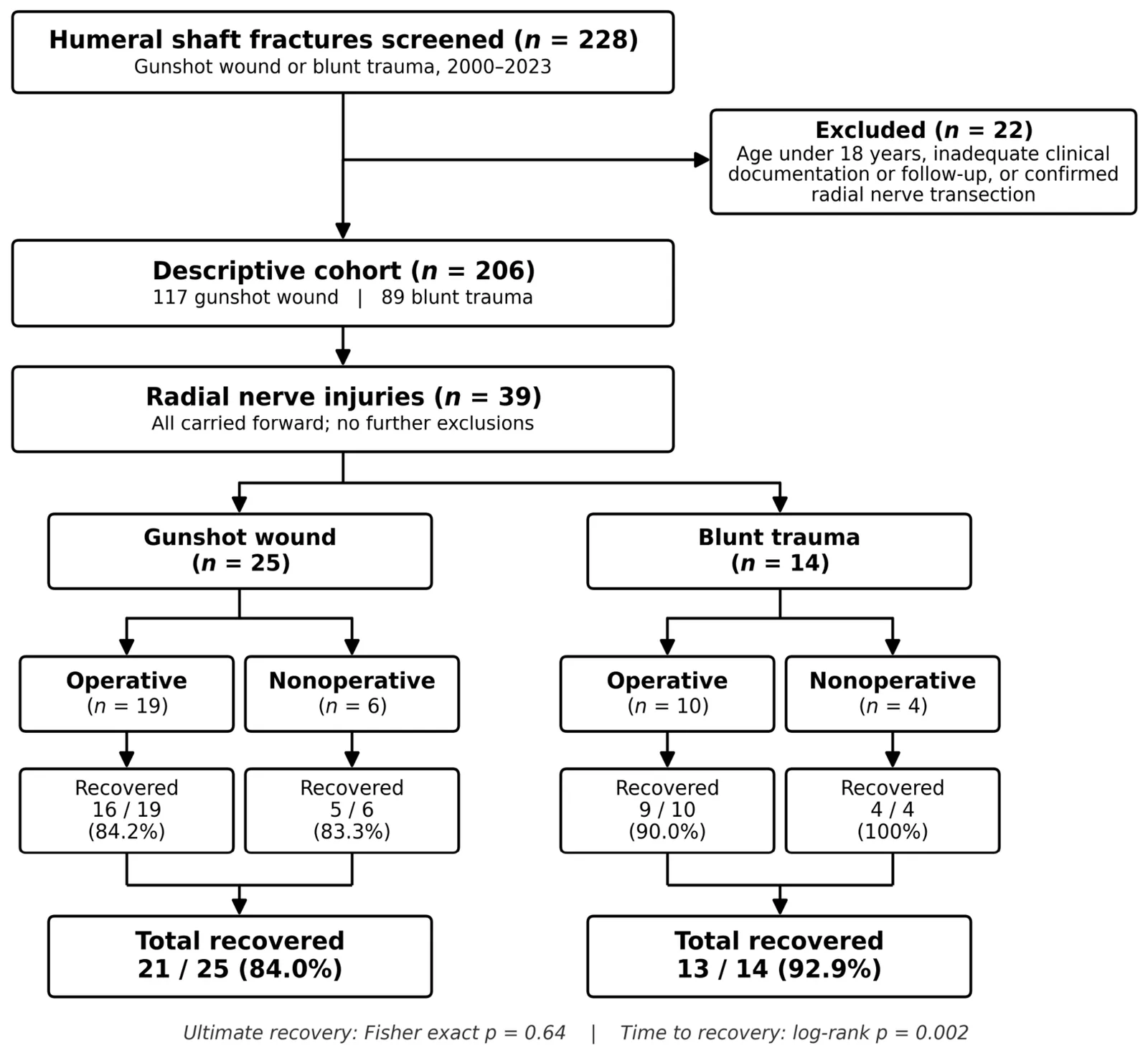
Study flow and derivation of the analytic cohort. Of 228 patients with humeral shaft fracture due to gunshot wound or blunt trauma identified between January 2000 and December 2023, 22 were excluded for age under 18 years, inadequate clinical documentation or follow-up, or confirmed radial nerve transection. This left a descriptive cohort of 206 patients (117 gunshot wound, 89 blunt trauma) containing 39 radial nerve injuries, all of which were carried forward without further exclusion: 25 after gunshot wound (19 operative, 6 nonoperative) and 14 after blunt trauma (10 operative, 4 nonoperative).

All 39 recorded radial nerve injuries were carried forward for analysis: 25 GSW (19 operative, 6 nonoperative) and 14 blunt (10 operative, 4 nonoperative) (Figure 1). Median follow-up was 8.2 months [IQR 5.3–14.9], with a maximum of 39.2 months. Follow-up was longer in the GSW arm (median of 11.9 [6.1–17.3] vs. 6.8 [4.5–8.0] months); however, because follow-up schedules were not standardized prospectively, this difference cannot be attributed definitively to differences in recovery time rather than surveillance intensity. Five patients had persistent radial nerve motor deficits without documented full recovery. These patients showed no meaningful clinical improvement during follow-up, with electrodiagnostic findings supporting a poor likelihood of further recovery, and were right-censored at their final neurologic assessment without a recovery event.

Full recovery was achieved in 34 of 39 patients (87.2%) and did not differ between mechanisms: 21 of 25 GSW (84.0%) versus 13 of 14 blunt (92.9%; *p* = 0.64). Within the operatively treated subgroup, recovery occurred in 16 of 19 GSW (84.2%) and 9 of 10 blunt patients (90.0%; *p* = 1.00). Outcomes by treatment strategy are summarized in Table 2 and Table 3. Among nonoperatively managed patients, five of six GSW patients recovered (83.3%) at a median of 5.3 months [5.2–6.7] and all four blunt patients recovered (100%) at a median of 3.4 months [1.9–5.1].

### 3.3. Primary Outcome: Time to Radial Nerve Recovery

**Figure 2:**
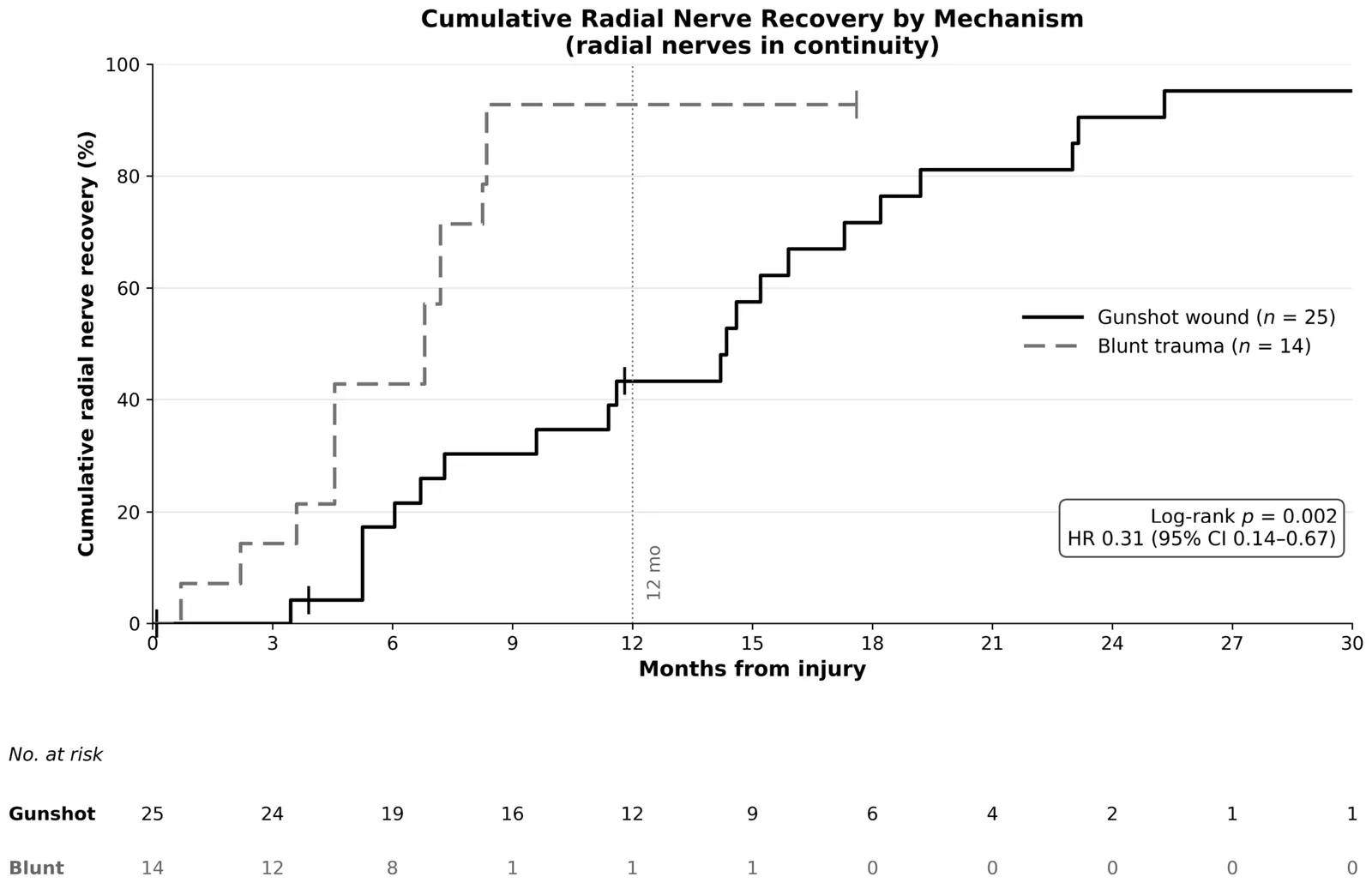
Kaplan–Meier estimates of cumulative radial nerve recovery by injury mechanism. Full recovery was defined as the first documented follow-up examination demonstrating restoration of full active radial nerve motor function, including extension of the forearm, wrist, and digits without residual motor deficit. Kaplan–Meier median recovery time represents the time at which the estimated cumulative probability of recovery reached 50%. Median recovery was 14.3 months after gunshot wound versus 6.9 months after blunt trauma (log-rank *p* = 0.002; HR 0.31, 95% CI 0.14–0.67). Tick marks indicate right-censored observations in patients who had not experienced the recovery endpoint at their final evaluable neurologic assessment. The dotted vertical line denotes 12 months. Numbers at risk are shown below the plot.

Recovery was significantly slower after ballistic injury. Kaplan–Meier median time to documented full recovery was 14.3 months (95% CI 7.5–17.3) after GSW versus 6.9 months (95% CI 3.6–8.2) after blunt trauma, a difference of approximately seven and a half months (log-rank *p* = 0.002) (Figure 2). In the Cox model, the GSW mechanism carried a hazard ratio for recovery of 0.31 (95% CI 0.14–0.67; *p* = 0.003), meaning that at any given time a patient with an in-continuity radial nerve injury after GSW was recovering at roughly 30% the instantaneous rate of a blunt trauma patient. The proportional hazards assumption was satisfied (scaled Schoenfeld residuals, *p* = 0.44), and restricted mean time to recovery over 24 months was 13.7 months after GSW versus 7.0 months after blunt injury (Table 4).

### 3.4. Confounder Stratification and Adjustment

Covariate distribution differed between arms in the directions expected. Vascular injury occurred only in the GSW arm (4/25, 16.0% vs. 0/14; *p* = 0.28) and distal fracture location was more common after GSW (56.0% vs. 42.9%; *p* = 0.51), both favouring the confounding hypothesis. Polytrauma, by contrast, was substantially more common in the blunt arm (92.9% vs. 44.0%; *p* = 0.005), favouring the opposite direction.

The primary inference rests on the unadjusted estimate (HR 0.31, 95% CI 0.14–0.67) and on the operatively confirmed sensitivity analysis reported in Section 3.6 (HR 0.27, 95% CI 0.11–0.69), both of which are stable and bounded well below unity. Single-covariate adjustment did not attenuate the mechanism effect in any model (Table 5). Given the substantial age difference between groups, a separate age-adjusted Cox model was also examined. Adjustment for age did not materially alter the association between injury mechanism and time to radial nerve recovery (HR 0.25, 95% CI 0.09–0.67; *p* = 0.006). Adjusting for vascular injury left the estimate essentially unchanged (HR 0.32, 95% CI 0.14–0.70; *p* = 0.005), and vascular injury itself carried no independent association with recovery (HR 0.83; *p* = 0.77). Adjusting for distal fracture location left the estimate essentially unchanged (HR 0.31, 95% CI 0.14–0.68; *p* = 0.004), as did adjustment for treatment strategy (HR 0.28, 95% CI 0.13–0.62; *p* = 0.002) and for sex (HR 0.32, 95% CI 0.14–0.70; *p* = 0.004). Two further models are reported as sensitivity analyses rather than as estimates of effect size. Polytrauma was present in 92.9% of the blunt arm and 44.0% of the GSW arm and is therefore close to collinear with mechanism, so the polytrauma-adjusted model (HR 0.19, 95% CI 0.08–0.47) and the exploratory model containing vascular injury, fracture location, and polytrauma simultaneously (HR 0.19, 95% CI 0.07–0.49) are numerically unstable; the latter carries roughly eight events per variable. These two models are reported to demonstrate that the mechanism effect does not reverse or attenuate under adjustment, not to quantify its magnitude. Across every specification the estimate remained bounded well below unity and never moved toward the null, which is the opposite of what confounding by associated injury severity would produce. Within strata, point estimates lay on the same side of unity in all estimable cohorts (Table 5). The smaller cohorts are underpowered, however: the non-distal fracture stratum (17 events) and the nonoperative stratum (9 events) are insignificant. With such few events, these stratified estimates are therefore reported descriptively and are not a basis for inference.

### 3.5. Landmark Recovery and the Timing of Late Recovery

Divergence between mechanisms was concentrated beyond one year. Cumulative recovery at 12 months was 43% after GSW versus 93% after blunt trauma; by 24 months the GSW curve had risen to 91% while the blunt curve remained at 93% (Table 4). The blunt cohort had essentially completed its recovery by one year, whereas the GSW cohort had not reached half of its eventual recovery at that point.

**Figure 3:**
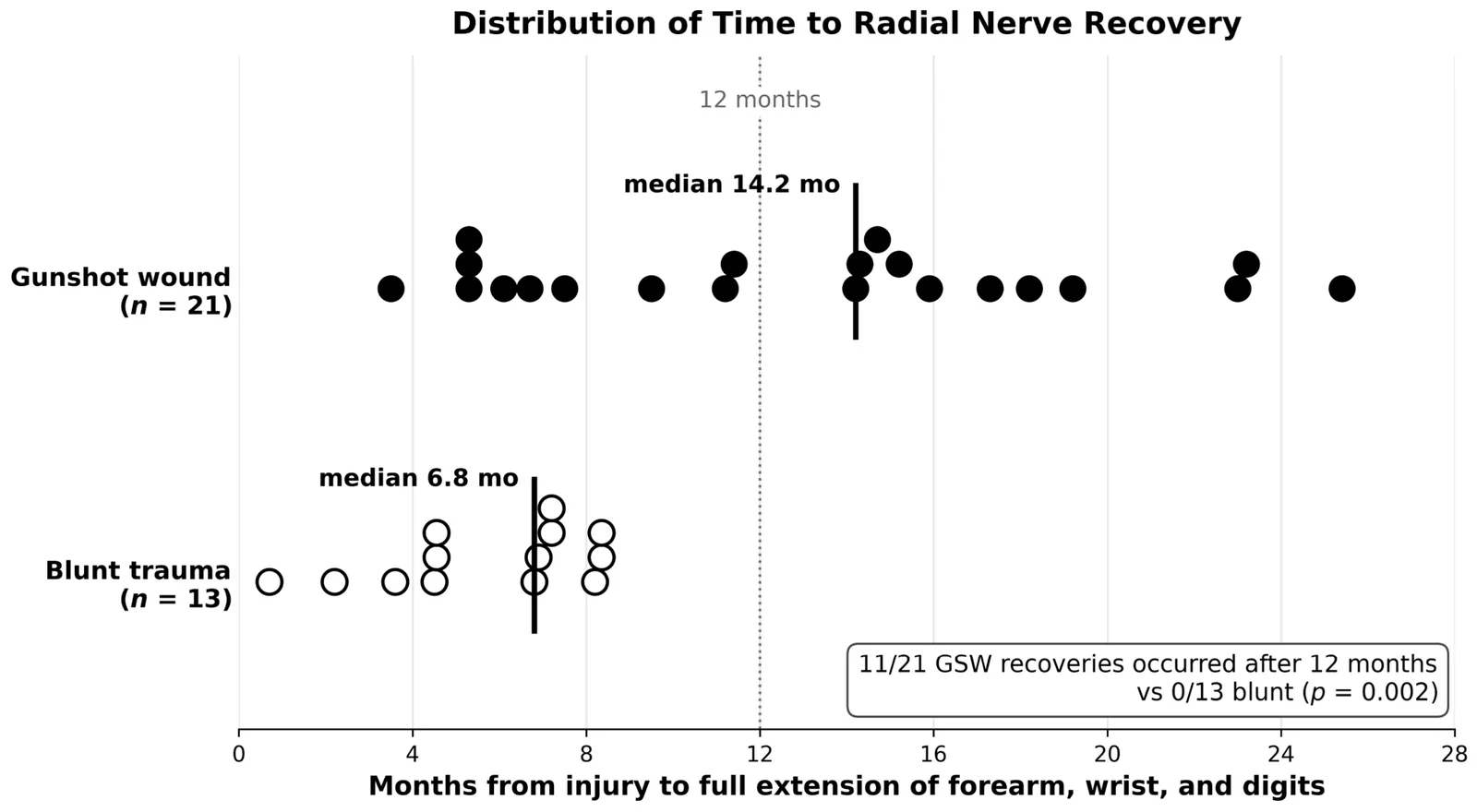
Distribution of observed time to full radial nerve recovery among patients who experienced the recovery endpoint. Each point represents one of the 34 patients with documented full recovery and is positioned according to the interval from injury to the first follow-up examination documenting full active extension of the forearm, wrist, and digits. Filled points represent GSW patients (*n* = 21) and open points represent blunt-trauma patients (*n* = 13); vertical solid bars indicate the observed median recovery times among recovered patients (14.2 months for GSW and 6.8 months for blunt trauma) and should not be interpreted as Kaplan–Meier medians. The dotted vertical line denotes 12 months. Eleven of 21 GSW recoveries occurred after 12 months compared with 0 of 13 blunt recoveries (Fisher exact *p* = 0.002).

Among the 34 patients who recovered, 11 of 21 GSW recoveries (52.4%) occurred more than 12 months after injury compared with 0 of 13 blunt recoveries (*p* = 0.002) (Figure 3). The latest blunt recovery occurred at 8.3 months; GSW recoveries were documented as late as 25.4 months.

### 3.6. Sensitivity Analyses

Two prespecified sensitivity analyses were performed. Restricting the cohort to the 29 patients in whom continuity was confirmed by direct operative inspection (19 GSW, 10 blunt; 25 recovery events) reproduced the primary result: Kaplan–Meier median time to recovery was 15.2 months after GSW versus 7.2 months after blunt trauma (log-rank *p* = 0.005), with a hazard ratio of 0.27 (95% CI 0.11–0.69; *p* = 0.006). The mechanism effect therefore does not depend on the ten patients in whom continuity was inferred rather than operatively confirmed. Penalized-likelihood (Firth) estimation, applied to address small-sample bias, gave a hazard ratio of 0.31 (95% CI 0.15–0.68; *p* = 0.004) in the full analytic cohort and 0.28 (95% CI 0.11–0.72; *p* = 0.009) in the operatively confirmed subgroup, in each case essentially identical to the corresponding maximum-likelihood estimate.

## 4. Discussion

Within a cohort restricted to radial nerves either surgically confirmed or clinically inferred to be in continuity, ballistic humeral shaft fractures were associated with significantly slower radial nerve recovery than blunt fractures, while the ultimate probability of recovery did not differ. The Kaplan–Meier median time to full extension of the forearm, wrist, and digits was roughly twice as long after GSW (14.3 vs. 6.9 months; *p* = 0.002) and the hazard of recovery at any moment was reduced by approximately 70% (HR 0.31). This is, to our knowledge, the first demonstration of a significant difference in radial nerve recovery timing between injury mechanisms.

### 4.1. Relationship to the Prior Cross-Mechanism Comparison

Radial neuropathy may also result from external compression, including so-called Saturday night, honeymoon, and crutch palsies. These injuries typically arise from focal compressive neuropathy rather than fracture-associated traction, displacement, or ballistic injury and are therefore outside the scope of the present comparison. The present study instead focused specifically on traumatic radial nerve injury associated with humeral shaft fractures.

Our finding does not contradict Guo and Chiou-Tan [16]; it addresses a different route of the same question. They compared what the lesions looked like, level of injury, completeness of injury, and associated nerve involvement, and found ballistic and blunt radial nerve injuries indistinguishable on all three [16]. We compared how the lesions behaved over time and found them clearly distinguishable. Lesions that appear equivalent on a single cross-sectional electrodiagnostic assessment nonetheless follow measurably different recovery trajectories.

### 4.2. Clinical Implications

Three practice points follow, within the limits noted below.

Patient counseling. After an in-continuity radial nerve injury from GSW, patients and surgeons should expect full extension of the forearm, wrist, and digits to take considerably longer than after blunt injury, a median of roughly 14 months, with about half of eventual recoveries occurring beyond one year. Counseling to a blunt-derived timeline risks premature conclusions of failure.

Electrodiagnostic testing. The 9-week threshold for nerve studies has been derived largely from blunt-trauma cohorts. Our findings demonstrate a longer recovery trajectory after ballistic injury, suggesting that mechanism-specific recovery timelines should be considered when interpreting serial clinical and electrodiagnostic assessments. However, this study did not directly evaluate the optimal timing of electrodiagnostic testing, and no specific testing interval can be established from these data alone [21].

Boundary of inference. The present study does not compare observation with exploration, nerve reconstruction, or tendon transfer and therefore cannot determine the optimal timing of surgical intervention or establish that surgery should be delayed. The findings instead suggest that recovery after ballistic radial nerve injury may continue over a longer interval than is typically described in blunt-trauma cohorts [14,15]. This longer recovery course should be considered during patient counseling and serial clinical assessment, but decisions regarding electrodiagnostic testing or surgical intervention must remain individualized and cannot be defined by these data alone. Reconstructive options such as nerve conduits or autologous nerve grafting may be appropriate for selected nerve-gap injuries, but these were outside the scope of the present study because surgically confirmed transections were excluded from the analytic cohort.

### 4.3. Confounding and the Limits of the Design

The most obvious objection to a mechanism effect is that it is not a mechanism effect at all, but a proxy for the established predictors of poor recovery that cluster with ballistic injury: transection, higher lesion grade, vascular injury. This study design addresses it at three levels.

Transection is removed by design rather than by adjustment. Surgically confirmed transections are excluded at screening, continuity is operatively confirmed in 29 of 39 patients, and no patient undergoes nerve repair or grafting. Vascular injury, fracture location, polytrauma, sex, and treatment strategy are addressed by stratification and single-covariate adjustment (Table 5), and the pattern of results is the opposite of what the confounding hypothesis predicts. If the mechanism effect is a proxy for associated injury severity, adjusting for those injuries should attenuate it toward unity and it does not. Adjustment for vascular injury leaves the estimate unchanged, and vascular injury carries no independent association with recovery in this cohort. Adjustment for distal fracture location, for polytrauma, and for all three severity covariates together each preserve the direction of the mechanism effect rather than moving it toward the null. Because polytrauma is nearly collinear with mechanism in this cohort, the point estimates from the polytrauma-adjusted and fully adjusted models are unstable and are not interpreted as effect sizes. Point estimates lie on the same side of unity in all estimable strata, though the smaller strata, non-distal fracture and nonoperative are underpowered and their confidence intervals include unity; they are reported descriptively and are not the basis for inference. The argument rests instead on the unadjusted estimate (HR 0.31, 95% CI 0.14–0.67) and the operatively confirmed sensitivity analysis (HR 0.27, 95% CI 0.11–0.69), both of which are stable and exclude unity. The stability of the mechanism estimate across these adjusted models suggests that the measured severity markers do not fully account for the observed association. However, residual confounding from incompletely measured or unmeasured factors cannot be excluded.

A similar argument applies to polytrauma, which was substantially more common in the blunt arm (92.9% versus 44.0%). Polytrauma would if anything be expected to slow recovery, so its concentration in the comparator arm biases against the observed effect, consistent with the finding that adjusting for polytrauma does not weaken the mechanism estimate. Because the two are nearly collinear, that adjustment is informative about direction only.

Lesion grade within the non-transected cohort remains an important potential confounder that could not be fully addressed. Although nerve conduction studies and MRI were used clinically in portions of the cohort to support assessment of nerve continuity, these studies were not applied to systematically characterize or grade initial lesion severity [22,23]. This limitation is shared by the entire prior literature, no previous study has stratified radial nerve recovery by lesion grade across mechanisms, and our design still removes the dominant severity confounder, transection, by construction, confirming continuity operatively in 29 of 39 patients. Guo and Chiou-Tan’s finding that completeness of injury does not differ between mechanisms is reassuring on this point, since it is direct evidence against the specific confounding pathway of concern, but it derives from a different cohort and cannot substitute for grading our own [16].

### 4.4. Limitations

This study is retrospective; operative fixation is indicated for neurovascular injury, polytrauma, or open fracture, raising the possibility of confounding by indication. The analytic cohort comprises 39 injuries with 34 events; the confidence interval around the hazard ratio (0.14–0.67) is comfortably bounded away from unity, though the covariate-stratified subgroups in Table 5 are smaller and some, particularly the distal-fracture stratum, where near-complete separation between arms precludes a stable point estimate, should be read as exploratory rather than definitive. The operative stratum reached significance when analyzed separately (log-rank *p* = 0.005), whereas the nonoperative stratum did not (*p* = 0.154). The nonoperative subgroup contained only ten patients and nine recovery events, resulting in limited statistical power and substantial uncertainty around the estimate. Although the point estimate remained in the same direction as the primary analysis, the subgroup result should therefore be interpreted cautiously. Continuity was inferred rather than operatively confirmed in the ten nonoperatively managed patients. Because electrodiagnostic studies were obtained non-uniformly during clinical care rather than by a standardized protocol, they could not be used to grade initial lesion severity consistently across the cohort; the recovery endpoint itself rests on serial clinical examination alone. One stratum could not be evaluated because it contained patients of one mechanism only: all four patients with vascular injury were GSW; the no-polytrauma stratum contained only a single blunt patient and, while nominally estimable, is too sparse on one side to be informative. Although time-to-event methods accommodate variable follow-up duration, they do not eliminate potential bias arising from differences in follow-up frequency. In addition, recovery was dated to the first clinical visit at which full recovery was documented; therefore, the true recovery date may have occurred between visits, resulting in some imprecision in the estimated time to recovery. Finally, this is a single-center experience in an urban US trauma system with predominantly low-velocity civilian ballistic wounds; recovery timelines should not be extrapolated to high-velocity military injuries, and the direct applicability of the findings is greatest where civilian firearm injury is common.

## 5. Conclusions

In this cohort of radial nerve injuries associated with humeral shaft fractures, documented full motor recovery occurred later after ballistic injury than after blunt trauma. Although full documented recovery rates were similar between groups, the small sample size and limited late follow-up warrant cautious interpretation of recovery probabilities. The longer recovery trajectory observed after ballistic injury should be considered during patient counseling and serial clinical follow-up, but these findings do not define an optimal duration of observation or timing of electrodiagnostic testing or surgical intervention.

## Figures and Tables

**Table 1:** Baseline characteristics of the descriptive cohort by injury mechanism. Percentages for the nested radial nerve rows are calculated within the radial nerve injury group. Age is summarized within each mechanism; IQR, interquartile range; WBC, white blood cell count. Fracture-location categories are not mutually exclusive; fractures spanning more than one anatomic region were counted in each involved category. Accordingly, fracture-location counts may sum to greater than the total cohort size.

Variable	Overall (*n* = 206)	GSW (*n* = 117)	Blunt (*n* = 89)	*p*-Value
Demographics				
Age (years), median [IQR]	—	28 [22–37]	44 [29–62]	<0.001
Male sex, *n* (%)	167 (81.1)	112 (95.7)	55 (61.8)	<0.001
Admission glucose (mg/dL)	123 [101–146]	125 [104–145]	122 [97–150]	0.797
Admission WBC (×10^3^/µL)	9.8 [7.4–13.0]	9.7 [7.6–12.9]	10.0 [6.9–13.9]	0.873
Injury and Treatment				
Operative treatment, *n* (%)	127 (61.7)	68 (58.1)	59 (66.3)	0.250
Polytrauma, *n* (%)	131 (63.6)	71 (60.7)	60 (67.4)	0.381
Any neurologic injury, *n* (%)	59 (28.6)	42 (35.9)	17 (19.1)	0.009
Radial nerve injury, *n* (%)	39 (18.9)	25 (21.4)	14 (15.7)	0.371
Operatively treated, *n* (%)	29 (74.4)	19 (76.0)	10 (71.4)	1.000
Nonoperatively treated, *n* (%)	10 (25.6)	6 (24.0)	4 (28.6)	1.000
Ulnar nerve injury, *n* (%)	16 (7.8)	12 (10.3)	4 (4.5)	0.188
Median nerve injury, *n* (%)	9 (4.4)	7 (6.0)	2 (2.2)	0.305
Vascular injury, *n* (%)	15 (7.3)	12 (10.3)	3 (3.4)	0.102
Fasciotomy, *n* (%)	10 (4.9)	7 (6.0)	3 (3.4)	0.520
Superficial infection, *n* (%)	14 (6.8)	8 (6.8)	6 (6.7)	1.000
Deep infection, *n* (%)	5 (2.4)	4 (3.4)	1 (1.1)	0.392
Nonunion, *n* (%)	6 (2.9)	4 (3.4)	2 (2.2)	0.700
Fracture Location				
Proximal shaft, *n* (%)	78 (37.9)	39 (33.3)	39 (43.8)	0.147
Midshaft, *n* (%)	56 (27.2)	31 (26.5)	25 (28.1)	0.875
Distal, *n* (%)	83 (40.3)	53 (45.3)	30 (33.7)	0.115

**Table 2:** Outcomes among operatively managed patients. Column headers give the full operatively treated cohort (*n* = 127). Rows above Radial Nerve Outcomes are calculated on those denominators; the radial nerve rows are calculated on the 29 radial nerve injuries within this cohort (19 GSW, 10 blunt), which are the denominators shown in those rows. Fixation-method categories were not mutually exclusive. Some patients underwent more than one fixation method during the course of treatment; therefore, fixation-method counts may exceed the number of operatively treated patients. * Mann–Whitney U test among patients who recovered. ** Log-rank test within the operatively treated subgroup. ORIF, open reduction and internal fixation.

Variable	GSW (*n* = 68)	Blunt (*n* = 59)	*p*-Value
Age (years), median [IQR]	29 [21–38]	39 [27–58]	<0.001
Male sex, *n* (%)	65 (95.6)	37 (62.7)	<0.001
Fixation Method			
ORIF, *n* (%)	55 (80.9)	46 (78.0)	0.826
Intramedullary nail, *n* (%)	6 (8.8)	2 (3.4)	0.283
External fixation, *n* (%)	9 (13.2)	10 (16.9)	0.623
Radial Nerve Outcomes			
Radial nerve injury, *n* (%)	19 (27.9)	10 (16.9)	0.203
Recovered, *n* (%)	16/19 (84.2)	9/10 (90.0)	1.000
Time to recovery (mo), median [IQR]	14.9 [11.0–18.5]	7.2 [4.6–8.2]	0.001 *
Kaplan–Meier median (mo)	15.2	7.2	0.005 **
Recoveries occurring >12 mo, *n* (%)	11/16 (68.8)	0/9 (0.0)	0.001
Other Outcomes			
Polytrauma, *n* (%)	40 (58.8)	42 (71.2)	0.193
Any neurologic injury, *n* (%)	31 (45.6)	12 (20.3)	0.003
Vascular injury, *n* (%)	10 (14.7)	3 (5.1)	0.086
Superficial infection, *n* (%)	4 (5.9)	5 (8.5)	0.732
Deep infection, *n* (%)	3 (4.4)	0 (0.0)	0.248
Nonunion, *n* (%)	3 (4.4)	2 (3.4)	1.000
Distal fracture, *n* (%)	39 (57.4)	26 (44.1)	0.157

**Table 3:** Outcomes among nonoperatively managed patients. Column headers give the full nonoperatively managed cohort (*n* = 79). Rows above Radial Nerve Outcomes are calculated on those denominators; the radial nerve rows are calculated on the ten radial nerve injuries within this cohort (six GSW, four blunt), which are the denominators shown in those rows. * Mann–Whitney U test among patients who recovered. ** Log-rank test.

Variable	GSW (*n* = 49)	Blunt (*n* = 30)	*p*-Value
Age (years), median [IQR]	27 [22–34]	49 [40–64]	<0.001
Male sex, *n* (%)	47 (95.9)	18 (60.0)	<0.001
Polytrauma, *n* (%)	31 (63.3)	18 (60.0)	0.814
Any neurologic injury, *n* (%)	11 (22.4)	5 (16.7)	0.580
Radial nerve injury, *n* (%)	6 (12.2)	4 (13.3)	1.000
Recovered, *n* (%)	5/6 (83.3)	4/4 (100.0)	1.000
Time to recovery (mo), median [IQR]	5.3 [5.2–6.7]	3.4 [1.9–5.1]	0.286 *
Kaplan–Meier median (mo)	6.7	2.2	0.154 **
Vascular injury, *n* (%)	2 (4.1)	0 (0.0)	0.523
Superficial infection, *n* (%)	4 (8.2)	1 (3.3)	0.644
Nonunion, *n* (%)	1 (2.0)	0 (0.0)	1.000
Proximal shaft, *n* (%)	23 (46.9)	20 (66.7)	0.107
Distal fracture, *n* (%)	14 (28.6)	4 (13.3)	0.168

**Table 4:** Time-to-event analysis of radial nerve recovery in the analytic cohort. A hazard ratio below 1.0 indicates slower recovery. Kaplan–Meier estimates were calculated using all 39 patients and incorporate censored observations. In contrast, observed time-to-recovery medians and interquartile ranges were calculated only among the 34 patients in whom full recovery was documented. Cumulative recovery percentages are Kaplan–Meier estimates. The comparison of recoveries occurring beyond 12 months is restricted to the 34 patients who recovered and were assessed using Fisher exact testing. Restricted mean time to recovery was calculated over a 24-month horizon and did not depend on the proportional hazards assumption. Late Kaplan–Meier estimates should be interpreted cautiously because few patients remained under observation at later time points. At 24 months, two GSW patients and no blunt-trauma patients remained at risk; the blunt Kaplan–Meier estimate remains unchanged from the last observed event rather than representing a newly observed 24-month follow-up.

Analysis	GSW (*n* = 25)	Blunt (*n* = 14)	Comparison
Primary Analysis			
Kaplan–Meier median, mo (95% CI)	14.3 (7.5–17.3)	6.9 (3.6–8.2)	Log-rank *p* = 0.002
Cox hazard ratio for recovery	0.31 (0.14–0.67)	Reference	*p* = 0.003
Cumulative Recovery (Kaplan–Meier)			
At 3 months	0%	14%	—
At 6 months	17%	43%	—
At 12 months	43.4% (95% CI 26.4–65.8), at risk 12	92.9% (95% CI 72.5–99.5), at risk 1	—
At 18 months	71.7% (95% CI 56.3–90.6), at risk 6	92.9% (95% CI 72.5–99.5), at risk 0	—
At 24 months	90.6% (95% CI 76.4–98.8), *n* at risk = 2	92.9% (95% CI 72.5–99.5), *n* at risk = 0	—
Supporting Analyses			
Recoveries occurring >12 mo, *n* (%)	11/21 (52.4)	0/13 (0.0)	*p* = 0.002
Restricted mean time to recovery, 24 mo	13.7 mo	7.0 mo	—
Observed time to recovery among patients who recovered, median [IQR]	14.2 [6.7–17.3]	6.8 [4.5–7.2]	*p* = 0.002
Full recovery, *n* (%)	21/25 (84.0)	13/14 (92.9)	*p* = 0.64
Model Diagnostics			
Proportional hazards (Schoenfeld)	—	—	*p* = 0.44

**Table 5:** Mechanism-stratified and covariate-adjusted estimates of the association between injury mechanism and time to radial nerve recovery. A hazard ratio below 1.0 indicates slower recovery after gunshot wound. Covariates are entered individually alongside mechanisms because 34 events cannot support a saturated model. No adjustment attenuated the mechanism effect toward unity. Point estimates lay on the same side of unity in all estimable strata, but the smaller strata were underpowered and their confidence intervals included unity, so the stratified estimates were descriptive rather than confirmatory. Vascular injury, distal fracture location, and polytrauma entered simultaneously; they are reported as exploratory (roughly eight events per variable). The operatively confirmed restriction and the operative-treatment stratum comprise the same 29 patients; the stratum row reports the log-rank *p*-value and the sensitivity row the Cox *p*-value. Firth penalized-likelihood estimation addresses small-sample bias. Strata in which the mechanism effect was not estimable or not stably estimable are reported in Supplementary Table S1.

Model/Stratum	Mechanism HR (95% CI)	*n* (Events)	*p*-Value
Unadjusted (primary)	0.31 (0.14–0.67)	39 (34)	0.003
Single-Covariate Adjustment			
+ Age	0.25 (0.09–0.67)	39 (34)	0.006
+ Vascular injury	0.32 (0.14–0.70)	39 (34)	0.005
+ Distal fracture location	0.31 (0.14–0.68)	39 (34)	0.004
+ Polytrauma	0.19 (0.08–0.47)	39 (34)	<0.001
+ Male sex	0.32 (0.14–0.70)	39 (34)	0.004
+ Operative treatment	0.28 (0.13–0.62)	39 (34)	0.002
+ All three severity covariates	0.19 (0.07–0.49)	39 (34)	<0.001
Mechanism Effect Within Strata			
No vascular injury	0.36 (0.17–0.79)	35 (31)	0.010
Non-distal fracture	0.53 (0.19–1.48)	19 (17)	0.22
Polytrauma	0.17 (0.05–0.55)	24 (21)	0.002
Operative treatment	0.27 (0.11–0.69)	29 (25)	0.005
Nonoperative treatment	0.37 (0.09–1.52)	10 (9)	0.154
Sensitivity Analyses			
Operatively confirmed continuity only	0.27 (0.11–0.69)	29 (25)	0.006
Firth penalized likelihood, full cohort	0.31 (0.15–0.68)	39 (34)	0.004
Firth penalized likelihood, operative only	0.28 (0.11–0.72)	29 (25)	0.009

## Data Availability

The de-identified data supporting the findings of this study are available from the corresponding author upon reasonable request. The data are not publicly available because they contain information that could compromise the privacy of research participants.

## Supplementary Materials

The following supporting information can be downloaded at: https://www.mdpi.com/article/10.3390/jcm15197603/s1, Table S1: Strata in which the mechanism effect could not be stably estimated.
