# Supplementary material for: Delayed Radial Nerve Recovery After Ballistic vs. Blunt Humeral Shaft Fractures: A Time-to-Recovery Analysis of Nerves in Continuity
Source: J Clin Med. 2026 Sep 30;15(19):7603. doi: 10.3390/jcm15197603 (PMC13666036; doi:10.3390/jcm15197603)
Supplement: Supplementary file 1 [file jcm-15-07603-s001.zip › jcm-4562818-supplementary.pdf]

Supplementary Material

Table S1. Strata in which the mechanism effect could not be stably estimated.

Stratum	Mechanism HR (95% CI)	n (events)	p-Value
Vascular injury present	Not estimable	4 (—)	—
Distal fracture	Not reported	20 (17)	<0.001
No polytrauma	0.48 (0.06–4.15)	15 (13)	0.50

These strata are reported for completeness and are not interpretable as effect estimates. The vascular injury stratum contained gunshot wound patients only. In the distal fracture stratum, near-complete temporal separation between arms renders the Cox point estimate numerically unstable, so the log-rank p-value is given instead. The no-polytrauma stratum contained a single blunt trauma patient (14 of 15 without polytrauma were gunshot wound) and is nominally computable but too imprecise to interpret. HR, hazard ratio; CI, confidence interval.
